# Short-term efficacy of intravitreal dobesilate in central serous chorioretinopathy

**DOI:** 10.1186/2047-783X-17-22

**Published:** 2012-07-12

**Authors:** Pedro Cuevas, Luis A Outeiriño, Carlos Azanza, Javier Angulo, Guillermo Giménez-Gallego

**Affiliations:** 1 Departamento de Investigación, IRYCIS, Hospital Universitario Ramón y Cajal, Ctra. de Colmenar, km. 9.100, Madrid -28034, Spain; 2 Departamento de Oftalmología, Hospital de Día Pío XII, Cuesta Sagrados Corazones 4, Madrid -28016, Spain; 3 Departamento de Estructura y Función de Proteínas, Centro de Investigaciones Biológicas, CSIC. C/ Ramiro de Maeztu 9, Madrid -28040, Spain

**Keywords:** Central serous chorioretinopathy, Fibroblast growth factor, Dobesilate, Intravitreal injection

## Abstract

**Purpose:**

To report the anatomic and functional outcome of intravitreal dobesilate to treat recurrent central serous chorioretinopathy (CSC).

**Methods:**

This is an interventional case report in which dobesilate was intravitreally injected in a case of recurrent CSC. Main measures included fundoscopy, Snellen visual acuity (VA) testing, fluorescein angiography and optical coherence tomography (OCT).

**Results:**

We present anatomical and functional evidences, obtained as early as eleven days after the treatment, of the efficacy of intravitreal dobesilate, in the treatment of chronic CSC condition. The effect after intravitreal dobesilate injection for CSC might be related to the normalization of retinal architecture.

**Conclusions:**

Intravitreal dobesilate may be an effective treatment option for recurrent CSC.

## Background

Central serous chorioretinopathy (CSC) is a well-characterized self-limiting disorder leading to serous neurosensory elevation of the retina. The acute form of the disease in many patients resolves spontaneously, with residual subjective impairment mainly in the form of faint scotomas or metamorphopsia [1]. Those patients who do not resolve spontaneously can develop chronic CSC with retinal pigment epithelium (RPE) and photoreceptor damage, resulting in permanent visual impairment.

The pathophysiology of CSC remains poorly understood. However, the cascade of events leading to neurosensory detachment includes, and may in fact begin with changes in choroidal permeability [2]. We recently reported that dobesilate, a well-characterized fibroblast growth factor (FGF) inhibitor [3] abolished vascular endothelial growth factor (VEGF)-driven vascular hyperpermeability and fluid leakage [4].

CSC was first described by von Graefe in 1866. CSC is a condition commonly seen in young or middle age adults as a localized detachment of the neurosensory retina in the macular region [5,6]. In acute CSC with focal leakage, RPE increases its function to absorb the subretinal fluid and the disease is self limiting. However, in those cases with persistent focal or chronic diffuse leakage, RPE may decompensate and thus gradually lead to a less favourable prognosis with visual loss [7]. Chronic CSC involves RPE detachment, macular pigmentary change, gravitational tract, teleangiectatic change of retinal capillaries, capillary nonperfusion, subretinal fibrosis, neuroretinal degeneration, and secondary choroidal neovascularization (CNV) [8,9]. CSC has a favourable natural course and typically results in spontaneous resolution of the detachment and improvement of visual function [10-12]. The high spontaneous remission rate favors conservative management as a first line therapeutic option. However, in some cases of CSC, patients may develop progressive visual loss resulting from persistent serous retinal detachment, cystoid macular degeneration or retinal pigment epithelium decompensation [6,11]. Thus, active intervention should be considered in CSC with a symptom duration lasting longer than 3 months [13,14] as it occurred with the eye included in the current study. Traditionally, the major treatment option for persistent CSC has included thermal laser photocoagulation [15,16]. However, focal laser treatment is not suitable for CSC with a subfoveal or juxtafoveal leaking point, and furthermore, possible complications of laser photocoagulation include CNV, conversion of metamorphopsia to scotoma and inadvertent foveal damage [17,18]. With the advent of indocyanine green angiography, it has been demonstrated that CSC primarily affects the choroidal circulation and causes multifocal areas of choroidal vascular permeability [8]. On the basis of indocyanine green angiographic findings, photodynamic therapy (PDT) with verteporfin has also been adapted for treatment of CSC [19]. However, photodynamic therapy can be associated with inflammation, fibrosis and subsequently with CNV [20,21] and is not specific enough in tackling the fundamental choroidal problem.

The endothelial cell barrier function is regulated by vascular endothelial tight junction proteins that are involved in the regulation of the movement of macromolecules through the endothelium. Modification of tight junction proteins by an increased concentration of VEGF directly results in elevated permeability and, as consequence, in tissue edema, in several pathological conditions, including cancer and neovascular diseases of the eye [22-24]. Inhibition of angiogenesis and vascular permeability can be an effective treatment for a variety of angiogenesis-dependent ocular diseases. Accordingly, it was proposed that VEGF antibodies could reduce choroidal hyperpermeability associated with CSC.

Treatment of acute and chronic forms of CSC with intravitreal injections of bevacizumab (Avastin) [25-28], a monoclonal antibody against VEGF, has, nevertheless, quite variable outcomes. Furthermore, frequent injections are normally required to achieve a final clinical stabilization [29]. Later on, several recent findings have additionally damped the initial enthusiasm for anti-VEGF treatment in ocular angiogenesis-dependent diseases, mainly in diabetic and age related macular cases. Although initially side-effects of anti-VEGF medications (endophthalmitis, rhegmatous retinal detachment, retinal tear, uveitis and vitreous hemorrhage) were supposed to be procedure- rather than medication-related, the large number of available data at this moment of the results of anti-VEGF therapies show that these treatment are accompanied of numerous unexpected side effects unrelated to the procedure used, including inflammation and fibrosis [24], development of RPE tears [30] and macular detachment [31]. In addition, other potential undesired off-target effects should be considered with chronic use of intravitreal anti-VEGF agents. Thus, the long-term inhibition of VEGF could adversely affect the health of neural retina, RPE and choriocapillaries, since these tissues constitutively express VEGF and rely on it for maintaining retinal health [32,33]. Accordingly, twenty percent of people with age-related macular degeneration (AMD) treated with anti-VEGF therapy have been shown to lose vision over time [34,35]. These caveats against the use of anti-VEGF therapy support the search of new efficient and safe therapies for ocular angiogenesis-related diseases.

Fibroblast growth factor (FGF) participates in CNV [36-39] which is a biological process associated with chronic CSC. The aim of the present interventional case was to find out whether intravitreal dobesilate, a specific FGF inhibitor [3] is a therapeutic option in the treatment of subretinal or intraretinal fluid accumulation secondary to chronic CSC. As FGF is a necessary mediator of VEGF activity, dobesilate also inhibits the last signaling network, as it has been recently reported [4].

## Case presentation

A 57-year-old Caucasian man presented at the Emergency Service because of onset of sudden blurred vision. He had suffered recurrent episodes of CSC for one year. The patient had received three consecutive monthly injections of Avastin with unsatisfactory results. Three months after the end of Avastin treatment, the patient was referred having had intense methamorphia in the right eye for the previous last five days.

## Methods

Ophthalmologic evaluations at baseline and 11 days after treatment included fundoscopy, Snellen visual acuity (VA) testing, fluorescein angiography and optical coherence tomography (OCT) through the dilated pupila. Central thickness was measured in OCT and defined as the distance between the internal limiting membrane and RPE, and included intraretinal fluid.

The eye was prepared in a standardized fashion, in compliance with the Helsinki Declaration. The patient received an intravitreal solution of dobesilate (150 μl) in his right eye under strict sterile conditions, following the International Guidelines for intravitreal injections [40].

Dobesilate was administered as a 12.5% solution of diethylammonium 2,5-dihydroxybenzenesulfonate (etamsylate; Dicynone Sanofi-Aventis). The pH of the solution was 3.2 at the opening of the phial and 5.2 after a 1:20 dilution in Milli-Q water. Mitogenesis experiments, carried out as described by Fernández *et al*. [3], show that etamsylate inhibits FGF-driven mitogenesis with the same efficiency as the potassium dobesilate salt employed in those studies, which first demonstrated this inhibitory activity (not shown).

After Ethical Committee approval from our Institution, informed consent was obtained from the patient after explanation of the nature and possible consequences of the study.

## Results

**Figure 1:**
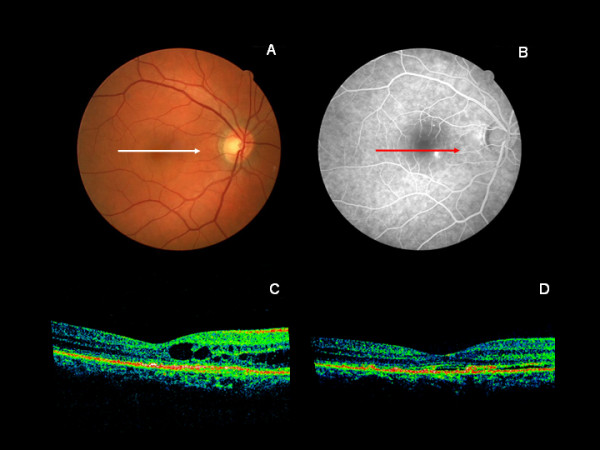
**Colour retinography at baseline** (**A**) Fluorescein angiography before dobesilate treatment (**B**) Spectral domain optical coherence tomogram (OCT), showing fluid-filled spaces in the area temporal to the fovea that distorts the normal architectural arrangement of the constituent cellular layer (**C**) The eye eleven days after receiving intravitreal injection of dobesilate, with marked resolution of intraretinal edema (**D**) Horizontal arrows indicate the localization of the OCT scans on the corresponding color fundus photography and fluorescein angiogram.

At baseline, colour retinography appeared normal (Figure 1A) and fluorescein angiography revealed a single small focal hyperfluorescent leak from RPE, the hallmark of CSC (Figure 1B). No diffuse degradation of the RPE was seen on the fluorescent angiogram. The OCT image depicted retinal cystoid abnormalities, and interstitial and subretinal fluid accumulation (Figure 1C). After dobesilate treatment, complete resolution of the intraretinal fluid was documented by OCT after eleven days follow-up (Figure 1D), with a corresponding visual improvement. At that point VA also showed an important improvement (0.4 at baseline vs. 0.8 after treatment), and there was an increase of four lines from baseline. Compared to baseline, retinal macular thickness (530 μm vs. 329 μm) and foveal retinal thickness (255 μm vs. 200 μm) had also decreased significantly. Choroidal detail revealed decreased vascular images, better seen in the area temporal to the fovea. No ocular or systemic side effects were observed. These effects were accompanied by focal fluorescent leak disappearance after treatment (not shown). The patient maintained this level of visual improvement during a month of follow-up.

## Discussion

Calcium dobesilate is the active principle of Doxium a drug used for more than 35 years. It has been orally administered for the treatment of diabetic retinopathy with a good safety profile [41]. Haritoglu *et al*. [42] have carried out a statistically sound study to assess the real clinical benefits of oral calcium dobesilate (Doxium) in the treatment of diabetic retinopathies. The study concluded that the oral administration of dobesilate did not show statistically significant clinical benefits. The discrepancy between these last results and those reported here may derive from the differences in the administration procedures. Oral administration is probably not the best choice for dobesilate to reach an adequate concentration at the vitreous. Dobesilate has a very low product of solubility at the acidic pH of the stomach, and further, readily oxidizes at the duodenal pH. Accordingly, local delivery seems a better choice in order to reach appropriate therapeutic concentrations of dobesilate in the case of well-delimited targets, as is the case reported here. Whether decreased leakage on fluorescein angiography indicates regression of new immature vessels, or whether it is simply a manifestation of the antipermeability effect of the drug, remains an open question. Recently, it has been reported that dobesilate abolished VEGF-induced vascular hyperpermeability [4]. This activity of dobesilate may account for the restoration of barrier malfunction in microvascular endothelial cells by normalization of tight junction proteins levels and organization [43].

The case we present suggests that dobesilate could be an efficient therapeutic agent in conditions of intraretinal fluid accumulation secondary to chronic CSC. Intravitreal dobesilate treatment led to significant reduction of central retinal thickness and significant gain in VA, and may improve the future management of CSC and other related blindness diseases. Obviously, the actual clinical value of dobesilate against CSC described here needs to be further investigated in a prospective randomized clinical trial with a longer follow-up. This study is ongoing.

## Conclusion

Intravitreal injection of dobesilate appears to lead to an improvement of visual acuity and neurosensory detachment, secondary to chronic serous chorioretinopathy, at least in the short term. Neither ocular toxicity nor adverse effects were observed. However, long-term studies are required with an adequate number of patients.

## Consent

Written informed consent was obtained from the patient for publication of this manuscript and accompanying images. A copy of the written consent is available for review by the Editor-in-chief on this journal.

## Abbreviations

AMD, Age-related macular degeneration; CNV, Choroidal neovascularization; CSC, Central serous chorioretinopathy; FGF, Fibroblast growth factor; OCT, Optical coherence tomography; PDT, Photodynamic therapy; RPE, Retinal pigment epithelium; VA, Visual acuity; VEGF, Vascular endothelial growth factor.

## Competing interests

The authors declare that they have no competing interest.

## Authors’ contributions

LAO performed patient treatment. LAO, CA and PC analyzed and interpreted patient data. PC and GGG are the major contributors to writing the manuscript, and in establishing the rational basis justifying the use of dobesilate and its form of administration in the case reported. JA supervised the manuscript. All authors read and approved the final manuscript.
